# Transcriptome dataset from bark and latex tissues of three *Hevea brasiliensis* clones

**DOI:** 10.1016/j.dib.2020.106188

**Published:** 2020-08-17

**Authors:** Mohd Fahmi Abu Bakar, Urwashi Kamerkar, Siti Nurfazilah Abdul Rahman, Muhd Khairul Luqman Muhd Sakaff, Ahmad Sofiman Othman

**Affiliations:** a Faculty of Bioresources & Food Industry, Universiti Sultan Zainal Abidin, Besut Campus, 22200 Besut, Terengganu, Malaysia; b School of Biological Sciences, Universiti Sains Malaysia, 11700 Minden, Pulau Pinang, Malaysia; c Centre of Chemical Biology, Universiti Sains Malaysia, 11900 Bayan Lepas, Penang, Malaysia

**Keywords:** *Hevea brasiliensis*, Transcriptome, Simple sequence repeats, Single nucleotide polymorphisms

## Abstract

*Hevea brasiliensis* is exploited for its latex production, and it is the only viable source of natural rubber worldwide. The demand for natural rubber remains high due its high-quality properties, which synthetic rubber cannot compete with. In this paper, we present transcriptomic data and analysis of three *H. brasiliensis* clones using tissue from latex and bark tissues collected from 10-year-old plant. The combined, assembled transcripts were mapped onto an *H. brasiliensis* draft genome. Gene ontology analysis showed that the most abundant transcripts related to molecular functions, followed by biological processes and cellular components. Simple sequence repeats (SSR) and single nucleotide polymorphisms (SNP) were also identified, and these can be useful for selection of parental and new clones in a breeding program. Data generated by RNA sequencing were deposited in the NCBI public repository under accession number PRJNA629890.

**Specifications Table**

**Table:** 

**Subject**	Biochemistry, Genetics and Molecular Biology (General)
**Specific subject area**	Genetics and Bioinformatics
**Type of data**	Table RNA Sequencing Dataset
**How data were acquired**	Next-generation sequencing using Illumina HiSeq 2000 platform
**Data format**	Raw Analysed
**Parameters for data collection**	Bark and latex tissues (RRIM 600, PB 260 and RRIM 929) was collected from 10-year-old rubber tree.
**Description of data collection**	Three rubber clones namely RRIM 600, PB 260 and RRIM 929 were used in this study, each with a low, medium, and high latex yield. From these clones, bark and latex tissue was collected from 10-year-old plants, with three replicates collected for each sample. Transcriptome analysis was performed using cDNA libraries of *H. brasiliensis*. The assembled contigs were also used for DEG analysis, as well as SNPs and SSRs marker discovery.
**Data source location**	Institution: PL Oil Palm & Rubber Sdn Bhd City/Town/Region: Kedah Country: Malaysia Latitude and longitude: 5.855802; 100.534269
**Data accessibility**	Repository name: GenBank (NCBI Sequence Read Archive) Data identification number: SRR11668401 - SRR11668418 Direct URL to data: https://www.ncbi.nlm.nih.gov/bioproject/PRJNA629890

**Value of the Data**

• These datasets are important to obtain the information from biological processes and pathways occur in the formation of natural rubber.
• These datasets can be useful for plant breeders to get the information for improving rubber breeding program as well as researchers which involved in the transcriptomic analysis and RNA dataset.
• The data will be of practical use in the development of genetic SNP and SSR markers as a tool in rubber breeding programmes.
• The data will facilitate further analysis aimed at identifying genes and pathways related to latex yield for the development of new rubber clone with improved performance.

## 1 Data description

The data presented here contain a combined transcriptome assembly of two different types of tissue (bark and latex) from three rubber clones (*Hevea brasiliensis*) collected in Malaysia (RRIM 600, PB 260 and RRIM 929). Rubber trees have been planted on a large scale in Malaysia since the 1920s. The transcriptome dataset generated from RNA-Seq can be used to identify high-expression genes that could be manipulated in order to improve desirable agronomy traits. Currently, there are more than 150 transcriptome datasets and bioproject of rubber tree available in the various online databases including NCBI (National Center of Biotechnology Information), EMBL (The European Bioinformatic Institute) and DDBJ (DNA Database of Japan). Previous transcriptome studies were focusing among others in phylogenetic relationships [1], latex flowing mechanisms [2] as well as molecular marker development involving single-nucleotide polymorphisms (SNPs) [3] and simple-sequence repeats (SSRs) [4], for rapid identification of parental and new clone characteristics. With the expansion of transcriptome dataset using three different rubber clones, the SNPs and SSRs markers associated with economic traits such as latex biosynthesis and disease resistance genes could be identified for improvement of new rubber clones traits during marker-assisted selection in rubber breeding program.

In this study, RNA isolation and the sequencing process were generated using the HiSeq 2000 platform. The raw reads generated was trimmed for quality analysis. RNA seq statistics from bark and latex tissues is shown in Table 1. A summary of the *H. brasiliensis* transcriptome analysis is shown in Table 2. Analysis showed that 46.5% of the transcriptome data had a significant match in the Swiss UniProt database; 26.7% in the KEGG database; 32.3% in the GO databases; and 39.4% in the Pfam database (Table 3). A summary of gene ontology (GO) annotation of the top three functions of transcripts found showed 187,573 transcripts related to molecular functions; 84,601 transcripts were biological processes; and 60,422 were cellular functions (Table 4). In this study, microsatellite motifs from merged transcriptomes were identified (Table 5), with dinucleotides being the most abundant, followed by trinucleotides, tetranucleotides and pentanucleotides. The number of SNPs within rubber transcriptome datasets is shown in Table 6, with the highest number of SNPs being identified from bark and latex tissue from the RRIM 929 rubber clone.

**Table 1:** RNA seq statistics of RRIM 600, PB 260 and RRIM 929 rubber clones from bark and latex tissues.

Specimens	Number of Raw Reads	Number of Clean Reads	Number of Bases (Gbp)	GC Content (%)
RRIM 600 Bark 1	321,079,316	284,939,062	32.4	48.2
RRIM 600 Bark 2	183,485,552	175,565,580	18.5	44.6
RRIM 600 Bark 3	409,017,628	357,973,318	32.2	48.3
RRIM 600 Latex 1	157,887,210	138,723,290	15.9	44.4
RRIM 600 Latex 2	188,628,236	153,984,894	19.1	43.9
RRIM 600 Latex 3	172,763,234	154,343,918	17.4	44.2
PB 260 Bark 1	140,597,008	126,049,224	18.6	44.6
PB 260 Bark 2	485,370,232	440,730,880	18.2	49.4
PB 260 Bark 3	137,787,002	118,730,722	16.6	46.8
PB 260 Latex 1	221,948,738	180,169,400	17.9	45.8
PB 260 Latex 2	126,340,176	112,311,382	19.4	46.5
PB 260 Latex 3	138,341,578	123,825,340	15.7	44.4
RRIM 929 Bark 1	184,515,844	174,777,904	14.2	44.0
RRIM 929 Bark 2	180,512,680	168,465,342	49.0	44.0
RRIM 929 Bark 3	164,214,400	155,141,574	13.9	44.1
RRIM 929 Latex 1	177,054,196	165,944,942	22.4	43.9
RRIM 929 Latex 2	192,012,738	180,594,218	12.8	45.2
RRIM 929 Latex 3	155,445,890	147,674,042	14.0	44.2

**Table 2:** Summary of *H. brasiliensis* transcriptome analysis.

Summary	Numbers
Total raw reads count	3737,001,658
Total clean reads count	3359,945,032
Number of transcripts	381,475
Maximum transcript length	48,514
Minimum transcript length	201
Mean transcript length	988
N50 length	1908
GC% content	39%

**Table 3:** Summary of the transcriptome annotations against several databases.

Databases	Number of Transcripts	Mapping Percentage (%)
Total number of transcripts	381,475	
UniProtKB/Swiss-Prot databases	177,454	46.5
KEGG	101,914	26.7
GO	123,291	32.3
Pfam	150,386	39.4

**Table 4:** Summary of gene ontology (GO) annotation analysis.

		Number of Transcripts
Annotated transcripts	GO-categorized	123,291
	Not GO-assigned	54,163
GO domains	Molecular functions	187,573
	Biological processes	84,601
	Cellular components	60,422

**Table 5:** Number of SSRs within rubber transcriptome datasets.

Motifs	Number of Contigs	
	100-bp flanking	50-bp flanking
Dinucleotide	10,993	12,767
Trinucleotide	1949	2172
Tetranucleotide	5	14
Pentanucleotide	3	3

**Table 6:** Number of SNPs within rubber transcriptome datasets.

Tissue	Number of SNPs		
	RRIM 600	RRIM 929	PB 260
Bark	85,188	168,666	101,776
Latex	129,947	151,057	74,295

## 2 Experimental design, materials and methods

### 2.1 Specimen collection

Bark and latex rubber-tree tissue was collected from a 10-year-old plant obtained from PL Oil Palm & Rubber Sdn Bhd (formerly known as Tradewinds Plantations Bhd), Kedah, Malaysia. Three replicates were collected for each tissue type.

### 2.2 RNA isolation and sequencing

The total RNA from the bark and latex tissue samples was extracted following the Qiagen RNAeasy Plant MiniKit protocol (Qiagen Inc., Chatsworth, CA). The resulting RNA quality and integrity was then estimated using standard Qubit Nanodrop spectrophotometry (O.D. ∼ 2.0) and Agilent 2100 Bioanalyzer (RIN value > 8) protocols. The paired-end Illumina mRNA libraries were generated using an Illumina TruSeq Kit from 1 ug of total RNA, following the manufacturer's protocol. Each sample was sequenced in multiple HiSeq 2000 lanes using the TruSeq SBS 36 Cycle Kit (Illumina, San Diego, CA) to obtain 2 × 101 bp reads.

### 2.3 Transcriptome analysis and mapping

Raw reads generated in FASTQ format obtained from Illumina platforms were analysed using FastQC, version 0.10.1 [5]. The raw reads were first screened for sequencing adaptors and then trimmed using Trimmomatic, version 0.32 [6]. The adaptor-trimmed raw sequences were then analysed for quality scores and bases with *Q* > 20. The sequences with Ns were removed before downstream analysis using Prinseq-Lite, version 0.2.0.4 [7]. Cleaned paired raw reads obtained in FASTQ format were mapped onto the draft *H. brasiliensis* genome [8] (accession: PRJDB4387) by Bowtie2, available from the DDBJ/EMBL/GenBank BioProject database, using TopHat software (version 2.1.0) [9]. Reads were mapped to the genome with default parameters. The mapped reads were then assembled using Cufflink v2.2.1 [9], with default parameters and selection of a minimum transcript length of 100 bp, to generate the reference transcriptome. The output transcripts were considered as *Hevea* reference sequences.

### 2.4 Transcriptome annotation

The clone transcripts were searched against UniProtKB/Swiss-Prot protein databases using BLASTX (version ncbi-blast-2.2.29+), with a cut-off e-value of 1e^−5^. The transcriptome analysis was annotated using the BLAST2GO program [10] and the gene ontologies (GO), Kyoto Encyclopaedia of Genes and Genomes (KEGG) and Pfam databases, on the basis of the BLASTX output.

### 2.5 Gene expression analysis

Expression profiling for each tissue sample was calculated according to the relative abundance of transcripts, aligned to the assembled reference sequence by Bowtie2 [11]. The expression levels of transcripts were calculated using Cufflinks (v2.2.1). The output was expected to show the value for each transcript with a 95% confidence interval.

### 2.6 Simple sequence repeat (SSR) and single-nucleotide polymorphism (SNP) discovery

Identification of contigs containing microsatellites (SSRs) was performed using the MISA program [12], and the minimum repeats were as follows: 10 for one base, six for two bases, and five for three, four, five and six bases; the interruptions (maximum difference between microsatellites) were 100 bases. Moreover, single-nucleotide polymorphism (SNP) calling was done using SAMtools, version 1.3.1 [13], to generate mpileup for one or multiple BAM files. VarScan version 2.3.9 [14] was used to perform SNP detection using default parameters. Reads were mapped against the reference transcriptome and filtered by determining which DNA bases were different from the reference. The putative SNPs selected were required to have a read depth equal to or greater than 10; the SNP reads/total reads ratio had to be equal to or greater than 0.25; the minimum phred score of bases had to be 20; and the SNP quality had to be 50.

## Ethics statement

This article does not contain any studies with human participants or animals performed by any of the authors.

## Declaration of Competing Interest

The authors declare that they have no known competing financial interests or personal relationships which have, or could be perceived to have, influenced the work reported in this article.

## Appendix A Supplementary materials

Supplementary Data S1 Supplementary Raw Research Data. This is open data under the CC BY license http://creativecommons.org/licenses/by/4.0/ Supplementary Data S1
